# A Digital Communication Assistance Tool (DCAT) to Obtain Medical History from Foreign-Language Patients: Development and Pilot Testing in a Primary Health Care Center for Refugees

**DOI:** 10.3390/ijerph17041368

**Published:** 2020-02-20

**Authors:** Frank Müller, Shivani Chandra, Ghefar Furaijat, Stefan Kruse, Alexandra Waligorski, Anne Simmenroth, Evelyn Kleinert

**Affiliations:** 1 Department of General Practice, University Medical Center Göttingen/Georg-August-University, Humboldtallee 38, 37073 Göttingen, Germany or stefan.kruse@stud.uni-goettingen.de (S.K.); evelyn.kleinert@med.uni-goettingen.de (E.K.); 2 Institute for Health System Solutions and Virtual Care (WIHV), Women’s College Hospital, 76 Grenville St, Toronto, ON M5S 1B2, Canada; Shivani.Chandra@wchospital.ca; 3 Crossroads Clinic, Women’s College Hospital, 76 Grenville St, Toronto, ON M5S 1B2, Canada; 4 Aidminutes GmbH, Bäckerstr. 6, 21244 Buchholz i. d. Nordheide, Germany; awaligorski@aidminutes.com; 5 Department of General Practice, University Medical Center Würzburg, Josef-Schneider-Straße 2, 97080 Würzburg, Germany; Simmenroth_A@ukw.de

**Keywords:** language barriers, app, interpreter, primary care, medical history taking, refugee, culturally and linguistically diverse patients

## Abstract

Background: Language barriers play a critical role in the treatment of migrant and refugee patients. In Germany, primary care interpreters are often not available especially in rural areas or if patients demand spontaneous or urgent consultations. *Methods:* In order to enable patients and their physicians to communicate effectively about the current illness history, we developed a digital communication assistance tool (DCAT) for 19 different languages and dialects. This paper reports the multidisciplinary process of the conceptual design and the iterative development of this cross-cultural user-centered application in an action-oriented approach. *Results:* We piloted our app with 36 refugee patients prior to a clinical study and used the results for further development. The acceptance and usability of the app by patients was high. *Conclusion:* Using digital tools for overcoming language barriers can be a feasible approach when providing health care to foreign-language patients.

## 1. Introduction

During the last decade, over 1.5 million asylum seekers migrated to Germany primarily due to increased persecution in the context of erupting civil war and poverty in Middle Eastern and African countries. The provision of primary health care to these groups of patients poses considerable challenges to both health care providers and the health care system. In order to provide adequate and cost-effective health care, overcoming language barriers between general practicioners (GPs) and patients is critical [1,2,3,4]. Researchers have not only underlined the importance of interpretation to overcome language barriers in medical encounters [3,5] but have also proven that having interpreters present during medical interactions will improve patient outcomes [6]. However, several studies revealed that providers consistently underuse professional interpreters even when they are readily available, despite understanding the benefits of having them present. Doctors try to “get by” because they assume that using interpreters will prolong and complicate consultations [7,8,9].

In every day practice, other barriers to using professional translators include challenges with quickly securing professional interpreters and unclear rules around who will cover the interpretation costs. Having family members interpret in a clinical setting is a commonly applied strategy, though studies have shown that medical translations by lay interpreters are often poor [10]. Additionally, the presence of a mistrusted lay or professional interpreter can lead to symptoms not being reported by patients and interpreters may inappropriately omit reasons for consultation that they consider to be “shameful” [11]. This can foster miscommunication between the physician and patient and contribute to an incomplete list of symptoms, ultimately leading to poor quality of care [12]. Although there are several tools to address this problem, including multilingual symptom reporting questionnaires, limited efforts have been made to develop digital tools for multilingual or intercultural settings [13,14,15,16,17,18,19].

## 2. Methods

An app-based multilingual digital communication assistance tool (DCAT) for refugee patients was developed to collect basic information about patients’ current health complaints and general medical history. The tool is meant to be used by the patient in the waiting room prior to seeing a healthcare provider. The DCAT guides patients through a query algorithm based on commonly stated reasons or health issues for medical appointments. After patients have answered the questions, a summary report is generated in German and is provided to the physician.

The intervention was developed using an action-oriented iterative approach with five phases: identification of the problem, reflection, planning, action, observation, and reflection [20]. This pragmatic approach allowed for the development of a complex intervention in this little understood area of work. The discursive development process involved experienced GPs, programmers, cultural scientists, sociologists, user interface (UX) designers, and numerous translators. The first step of the development process was to create a query algorithm based largely on an approach developed by Braun, a physician who used 82 diagnostic protocols to thoroughly record various health complaints [21,22]. A team of experienced GPs identified areas of Braun’s diagnostic protocols that could be obtained by asking the patient directly, without having to conduct a clinical examination or other medical tests. Subsequently, these protocol excerpts were organized into symptom categories and the information to be queried was converted into patient-facing questions with corresponding answer options. The entire project was led by GPs in a discursive process, leveraging German and European primary care guidelines, external literature, and personal experiences [23]. Questions were first transferred into plain language and then translated into several languages by professional interpreters. Once complete, these translations were recorded as audio or video files. UX designers converted the query structure into an app design, which was ultimately programmed by software developers. For evaluation purposes, DCAT was able to generate a detailed report about the health issues that were input by the user. These reasons for the patient’s visit were coded by DCAT using the International Classification of Primary Care (ICPC-2) [24].

DCAT was primarily designed for an iOS environment, and Apple iPad Mini 4 devices were used to administer the app. After preliminary testing with foreign-language colleagues, a three-week testing phase was supported by intensive field observation and technical backup in an initial reception facility for refugees. More details about the implementation process and test field are available in the Furaijat [16]. This study received approval from the Medical Ethics Committee of the University Medical Center Göttingen (Ethics Approval No. 16/3/17) and is registered in the German Clinical Trials Register (DRKS00013076).

## 3. Functions and Contents of the App

The DCAT app includes the most common health complaints and reasons for medical consultation from which patients can select one or more options (Table 1). Patients were asked to sort the symptoms or complaints according to their subjective feeling of severity. Then, several branching questions followed each reported symptom (e.g., "When did the complaint start?”, “Were you already in medical treatment because of this complaint?", etc.). Questions can be answered by ticking checkboxes and activating toggles. In-app tutorials support the patients with learning and using the interface, and audio and video supports allow illiterate patients to use the application without full knowledge of written language. Entering free text was not considered in the current version because it requires patients’ extensive understanding of written language, the ability to use a touchscreen keyboard, and poses challenges for instant translation when using dialectal expressions. During our testing phase, pictograms were often misunderstood and their use in the intervention was reduced to a minimum.

A total of 1800 different items (e.g., questions and answer options) can be selected and allow patients to provide a detailed description of their symptoms, health concerns, and the perceived severity of these items. Culturally relevant conceptions of symptoms were used to describe these various health concerns. The information entered by patients triggers further branching questions (e.g., on alcohol and drug consumption, previous illnesses, previous surgery, or current drug treatment). Thus, DCAT adaptively reacts to patients’ given information and appears more flexible to users than a standard questionnaire.

In its current version, DCAT supports 19 different languages and dialects (Table 2). For every question, control element, and answer option, the tool offers an audio or video output in the corresponding language or dialect. This means that users do not have to read any text to use the app and can rely solely on the audio or video features of the app to use it successfully. For many languages, users also have the option of selecting whether they would prefer to have a female or male voice guide them through DCAT.

A unique feature of the intervention is that it not only supports standardized languages but also allows users to select from a variety of widely used colloquial dialects for which no codified written languages exist. Therefore, these commonly used dialects represent the actual vocabulary of patients used for the expression of topics such as intimate issues concerning health, psychological wellbeing, or sexuality. Arabic dialects are a good example: the mostly written Modern Standard Arabic is understood as a lingua franca by a fraction of the Arabic-speaking population, whereas the various locally used Arabic dialects form the actual spoken language in everyday life and depend on the individual’s country of origin and level of education. DCAT uses plain language wherever possible to ensure that patients clearly understand the questions and provide their healthcare provider with correct information about their health complaints.

Translators were advised to find the most common, easy to grasp, and comprehensive syntax. Linguistic translations without direct language equivalents for body parts posed a particular challenge. For example, we found that in some languages, certain descriptions of the body were of a technical-anatomical nature, which were uncommon and therefore difficult to understand for people with little or no education. However, any commonly understood alternative translation would use vulgar and thus an inappropriate term. In these cases, the interdisciplinary team created adequate paraphrases that were medically accurate and understandable as well as appropriate to the medical setting. We developed a 3D body model where the patients can rotate the figure and where applicable, indicate the exact area of discomfort. This was introduced to avoid misunderstandings, particularly for languages with limited options to describe some areas of the body with precision.

**Figure 1:**
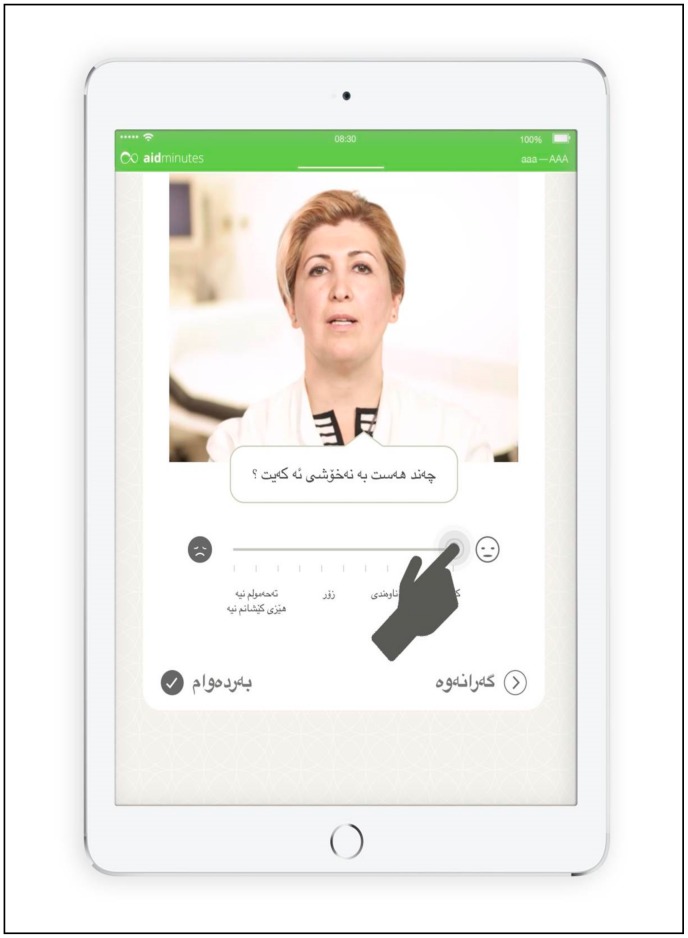
In-app tutorial in the Kurdish Sorani version showing how to use the analog scale control. In the video the user is asked: “How severe are your complaints?” The slider element, as well as the proceed and backward buttons, are adapted to the reading direction (right to left).

Lastly, we paid significant attention to the design of the user interface. To meet the requirements of language reading direction, the menu navigation changes according to the reading direction of the language or the contrary orientation of the analog scale used to indicate the intensity of pain (Figure 1). In order to provide a user experience that builds upon previous treatment experiences with physicians, the structure and flow of the questions were modeled on how a physician consultation would typically occur. Additionally, questions are presented by pre-recorded videos with interpreters who are “performing” a doctor role. Unlike typically rigid questionnaires, these videos were effective at attracting patients’ attention and interest.

## 4. Results of Pilot Testing

We conducted the intervention’s pilot test with 36 patients in the primary health care center of a reception center for asylum seekers in Friedland, Germany (Grenzdurchgangslager Friedland). In this health care center, medical professionals did not have access to professional in-person or phone language translation services. The majority of the patients were of Syrian origin (N = 20), had a median age of 31.5 years, and more than half (N = 19) were female. The median duration of DCAT use was 12:51 minutes (min: 6, max: 47 minutes). During this testing phase, between 5 and 12 symptoms (median: 8) were recorded per patient according to ICPC, with an average time of 1.7 minutes per symptom. The most frequent main complaints selected by the patients were flu (N = 8), cough (N = 5), and stomach pain (N = 5). Arabic Levante (N = 13) was the most commonly selected language for translation, followed by Modern Standard Arabic (N = 8), Arabic Egyptian (N = 5), Farsi/Persian (N = 5), Kurdish Sorani (N = 3), and Turkish (N = 2). More than half of the patients used DCAT’s audio support. Upon completion of the questions, patients were asked in a digital and audio supported questionnaire whether they were able to use DCAT well (yes/partially/no) and if they felt they could input their essential medical information (yes/partially/no). The results are shown in Table 3.

## 5. Discussion

This technology is meant to improve quality of care experienced by foreign-language speakers, including refugee newcomers and other communities that may be particularly likely to experience care access barriers. Navigating health systems and accessing medical care can be extremely challenging for patients who are unable to effectively communicate with their healthcare provider. When interpretation services are not present, patients are more likely to have a negative healthcare experience and receive incomplete medical care. This tool is meant help clinical care providers and help reduce these inequities by improving communication and care for newcomers and foreign-language speakers.

Various studies have shown that doctors often avoid consultations with interpreter support because they expect them to lengthen and complicate the appointment. Additionally, the cost of using in-person or live video interpreters creates additional access barriers for medical professionals to request their services due to limited funding. Typically, it is the medical professional who will decide if an interpreter will be used [7]. Studies have shown that doctors often overestimate the language competency of their patients [25,26,27] and are more likely to decide against requesting an interpreter.

DCAT attempts to fill this gap; GPs can rely on accurate translations, information is received directly from patients, and confidential documentation can be obtained without relying on a third person. Patients are able to have a more independent healthcare experience without having to rely on others, including women who must ask their family members to serve as a lay interpreter when questions of a sexually intimate nature are being asked by the provider. By using DCAT before the consultation in a waiting room, the actual appointment is not disturbed or prolonged. This also distinguishes our approach from those which introduce a digital ad-hoc translation (e.g., using Google translate during the consultation) [28,29,30] and apps that only allow health care providers to communicate preselected phrases and are therefore not self-administered by patients [14,15,17,18]. Furthermore, the DCAT app is free to download, making it accessible to all clinics that have a digital device they can circulate. Lastly, patients who have basic language skills for everyday life but who may lack the advanced fluency to accurately describe specific medical concerns can also use the intervention.

In our pilot study, DCAT was used in a clinical situation where an interpreter service was not available. Although DCAT thus served as a substitute for interpreters, it is likely that DCAT can also be a useful supplement for consultations when patients are accompanied by professional or lay interpreters. With the DCAT information available before the consultation, patients can be triaged and medical professionals might be better prepared for meeting patients’ needs and expectations. As DCAT does not provide a direct “backchannel”, interpreters are also necessary to explain treatments and medication individually. Nonetheless, the approach needs further adjustment. Although only a minority of respondents reported that their complaints were not included in DCAT, these outlying reasons for consultation will be considered in future iterations of the app. These specific complaints are normally treated by specialists; however, because primary care is the point of entry for many into the healthcare system, having a greater breadth of health complaints included in DCAT will be considered. Also, our study focused mainly on patients’ experiences with DCAT and excluded the perspective of health care providers. However, a key aspect of an upcoming study will examine how doctors and nurses in general practices perceive DCAT and which obstacles may arise in implementing it in the daily work routine.

Using digital communication assistance tools for overcoming language barriers is also applicable in other fields of health care. In the follow-up project, DICTUM Rescue, the DCAT approach will be applied in paramedic care, a field where it is rare to have external interpreters present. Also, DCAT might be useful for triage patients when they seek care outside of scheduled appointments (e.g., in an emergency room or walk-in clinic).

## 6. Conclusions

The development of our digital communication assistant tool was a highly complex process, especially when considering the aspects of accessibility as well as cultural and language sensitivity. Our initial findings are very promising. In the first pilot phase, our tool showed that it was widely accepted and effectively used by refugee patients. Based on the initial findings of this study, we suggest that the DCAT app be used to compliment live interpretation during a clinical visit. Future studies will explore specific recommendations on how best to incorporate the DCAT into clinical care. Whether clinical outcomes can also be improved will be examined in further studies.

## Figures and Tables

**Table 1:** Main complaints, symptoms, and reasons for consultation.

Complaints		
Stomach pains	Lower abdominal pain	Leg pain
Chest pain/heart ailments	Palpitations	Bone fracture or severe bruise
Paralysis or numbness	Common cold	Flu
Fever	Cough	Difficulty swallowing
Diarrhea	Change in bowel movements	Vomiting or nausea
Earaches	Unaccounted for sounds e.g., ringing in the ears or hearing voices	Rash
Allergy	Insect bite or sting	Sleep disorder
Injury	Wound	Bleeding
Burn	Fainting or blacking out	Weight loss
Dizziness	Weakness	Shortness of breath
Depression	Sore Throat	Headaches
Back Pain		

**Table 2:** Supported languages.

Supported Languages		
German ^1^	English ^1^	French
Arabic MSA	Kurdish Kurmanji	Turkish
Arabic Levante	Kurdish Sorani	Russian
Arabic Egyptian	Farsi (Persian)	Polish
Arabic West Maghreb	Lithuanian	Spanish
Arabic East Maghreb	Dutch	Italian
Pashtu		

^1^ available output languages of the summary report.

**Table 3:** Patients’ assessment after using the digital communication assistance tool (DCAT).

Question	Yes *N* (%)	Partially *N* (%)	No *N* (%)
**Were you able to use DCAT well?**	29 (80.6)	6 (16.7)	1 (2.8)
**Were you able to enter your essential information?** ^1^	26 (72.2)	0 (0)	3 (8.3)

^1^ Missing = 7.
